# Hemeoxygenase-1 Mediates an Adaptive Response to Spermidine-Induced Cell Death in Human Endothelial Cells

**DOI:** 10.1155/2013/238734

**Published:** 2013-08-04

**Authors:** Hana Yang, Seung Eun Lee, Gun-Dong Kim, Hye Rim Park, Yong Seek Park

**Affiliations:** Department of Microbiology, School of Medicine, Kyung Hee University, No. 1 Hoegi-Dong, Dongdaemun-Gu, Seoul 130-701, Republic of Korea

## Abstract

Spermidine (SPD) is a ubiquitous polycation that is commonly distributed in living organisms. Intracellular levels of SPD are tightly regulated, and SPD controls cell proliferation and death. However, SPD undergoes oxidation in the presence of serum, producing aldehydes, hydrogen peroxide, and ammonia, which exert cytotoxic effect on cells. Hemeoxygenase-1 (HO-1) is thought to have a protective effect against oxidative stress. Upregulation of HO-1 in endothelial cells is considered to be beneficial in the cardiovascular disease. In the present study, we demonstrate that the ubiquitous polyamine, SPD, induces HO-1 in human umbilical vein endothelial cells (HUVECs). SPD-induced HO-1 expression was examined by Western blot and reverse transcription-polymerase chain reaction (RT-PCR). Involvement of reactive oxygen species, serum amine oxidase, PI3K/Akt signaling pathway, and transcription factor Nrf2 in the induction of HO-1 by SPD was also investigated. Furthermore, small interfering RNA knockdown of Nrf2 or HO-1 and treatment with the specific HO-1 inhibitor ZnPP exhibited a noteworthy increase of death of SPD-stimulated HUVECs. In conclusion, these results suggest that SPD induces PI3K/Akt-Nrf2-mediated HO-1 expression in human endothelial cells, which may have a role in cytoprotection of the cells against oxidative stress-induced death.

## 1. Introduction

Polyamines are common cell components among almost all types of organisms, which regulate cell proliferation and differentiation. The function of polyamines has been studied at the molecular level. Polyamines modulate the function of RNA, DNA, and protein by promoting their stability and synthesis [1]. Polyamine content within cells is tightly regulated and retained both by polyamine biosynthesis and by polyamine transport according to needs [2]. For example, it is reported that proliferative stimuli, such as DNA synthesis, trigger uptake and/or biosynthesis of polyamines [3]. There are three polyamines in eukaryotic cells: putrescine, spermidine, and spermine. As long as the polyamine pool is strictly regulated also by interconversion, excessive polyamine accumulation following exogenous addition may exert a different effect on cells. Extracellular polyamines first undergo oxidation by serum amine oxidase, producing aminoaldehydes, such as aminomonoaldehyde [*N*′-(4-aminobutyl)-aminopropionaldehyde] and a dialdehyde [*NN*′-bis-(3-propionaldehyde)-1,4-diaminobutane] [4]. These aminoaldehydes and other oxidation products (e.g., H_2_O_2_ and NH_3_) show cytotoxicity in several cell types [5, 6]. Due to the oxidation by serum amine oxidase, the endothelium would be the first tissue with which the products of polyamine oxidation interact. 

Vascular injury generally refers to structural and functional impairment of endothelium including damage at the cellular level. It plays a key role in the pathogenesis of various cardiovascular diseases, such as atherosclerosis, diabetic complications, and hypertension. Hemeoxygenase (HO) is one of the cytoprotective proteins that could confer a beneficial effect in vasculature. HO originally functions as a rate-limiting enzyme in heme degradation, yielding carbon monoxide (CO), iron, and biliverdin as the end products. HO-1, HO-2, and HO-3 are isoforms of HO in mammals [7, 8]. In particular, the role of hemeoxygenase-1 (HO-1) as a protective enzyme is well known. Its antioxidant, antiapoptotic, and anti-inflammatory effects have been extensively studied [9, 10]. HO-1 is a stress-inducible protein. Various stimuli, such as thiol scavengers, ultraviolet radiation, and oxidative stress, lead to HO-1 upregulation. Usually, these types of stimuli generate reactive oxygen species (ROS), which may cause an adaptive response to HO-1. HO-1 is one of the phase II detoxifying and antioxidant enzymes that are modulated by the nuclear factor-erythroid 2-related factor 2 (Nrf2)/Kelch-like ECH associating protein 1 (Keap1)/antioxidant responsive element (ARE) transcription factor system [11]. Inhibition of Keap1 activity allows Nrf2 translocation from cytoplasm to nucleus, and then Nrf2 binds to the ARE region to activate expression of phase II gene, such as HO-1 [12]. Upregulation of HO-1 has protective effects in various cell types due to the activities of its final products, such as CO and/or bilirubin [13, 14]. HO-1 is also known to exert a beneficial effect in several clinically relevant diseases, especially vascular disease, including atherosclerosis, diabetes, and hypertension [15]. HO-1, which is highly expressed in vascular tissues under certain conditions, protects against cardiovascular diseases and contributes to sustain the health of the vascular system. In human endothelial cells, lack of HO-1 leads to endothelial damage induced by tumor necrosis factor-*α* and interleukin-1*α* [16]. These results suggest that HO-1 may play an important role in the human cardiovascular system.

In this study, we demonstrate that polyamine SPD induces HO-1 expression in human endothelial cells and the association of serum amine oxidase, ROS, and phosphatidylinositol 3-kinase (PI3K)/Akt-Nrf2-ARE signaling pathway in the upregulation of HO-1 by SPD.

## 2. Materials and Methods

### 2.1. Materials

M199 medium and fetal bovine serum (FBS) were purchased from WELGENE (Daegu, Korea). TRIzol reagent was supplied by Invitrogen (Carlsbad, CA, USA). Spermidine (SPD), aminoguanidine (AG), N-acetyl cysteine (NAC), MTT [3-(4,5-dimethylthiazol-2-yl)-2,5-diphenyltetrazoliumbromide], and dimethylsulphoxide (DMSO) were provided by Sigma Chemical (St. Louis, MO, USA). ExGen 500 reagent was obtained from Fermentas (Hanover, MD, USA). Anti-Nrf2 and anti-Lamin B antibodies were purchased from Santa Cruz Biotechnology (Santa Cruz, CA, USA). Anti-HO-1 antibody was obtained from Epitomics (Burlingame, CA, USA). Anti-GAPDH antibody was supplied by AbFrontier (Seoul, Korea). All other chemicals and reagents were of analytical grade.

### 2.2. Cell Culture

HUVECs were maintained in M199 medium and supplemented with 10% fetal bovine serum, 1% penicillin and streptomycin, 10 ng/mL human fibroblast growth factor, and 18 mU/mL heparin. The cells were incubated at 37°C under 5% CO_2_ atmosphere. HUVECs were grown to approximately 80% confluence, maintained with the fresh medium previously described, and subcultured every 2-3 days [17]. The cells were used within passages 4–9 during these experiments. 

### 2.3. Western Blot Analysis

We applied 20 *μ*g of the whole cell lysate proteins to each lane and analyzed them with Western blot. Western blot analysis was performed using monoclonal antibody against hemeoxygenase-1 (HO-1), anti-Nrf2, anti-Lamin B and anti-glyceraldehyde-3-phosphate dehydrogenase (GAPDH). Horseradish peroxidase-conjugated anti-IgG antibodies were used as the secondary antibodies to detect the previously mentioned protein bands by enhanced chemiluminescence WESTSAVE-Up (AbFrontier, Seoul, Korea) [18].

### 2.4. RNA Isolation and Reverse Transcriptase-Polymerase Chain Reaction (RT-PCR)

Reverse transcription was performed as previously described [19]. The primer sequences for human HO-1 were 5′-ACATCTATGTGGCCCTGGAG-3′ (forward) and 5′-TGTTGGGGAAGGTGAAGAAG-3′ (reverse). The amplified products were resolved by 1% agarose gel electrophoresis, stained with ethidium bromide, and photographed under ultraviolet light. 

### 2.5. Measurement of ARE Promoter Activity

EpRE/ARE-luciferase (EpRE/ARE-Luc) reporter plasmid was a generous gift from Dr. R. K. Park, (Wonkwang University, Korea). EpRE/ARE-Luc was generated by transfer of the enhancer 2 (E2) and minimal promoter (MP) sequences into the luciferase reporter plasmid pGL3-Basic. ARE promoter activity was determined as previously described [20].

### 2.6. Terminal Deoxynucleotidyl Transferase-Mediated dUTP Nick-End Labeling (TUNEL) Assay

To measure DNA fragmentation, the commercially available *in situ* death detection kit (Roche Diagnostics, Mannheim, Germany) was used. The cells were examined by fluorescence microscopy and photographed as previously described [21]. TUNEL-positive cells were determined by counting at least 100 cells in 3 randomly chosen fields and by expressing them as a percentage of the total number of cells counted.

### 2.7. Statistical Analysis

Statistical significance was estimated by Student's *t*-test, and the results were expressed as mean ± SD.

## 3. Results

### 3.1. HO-1 Induction by SPD in HUVECs

**Figure 1:**
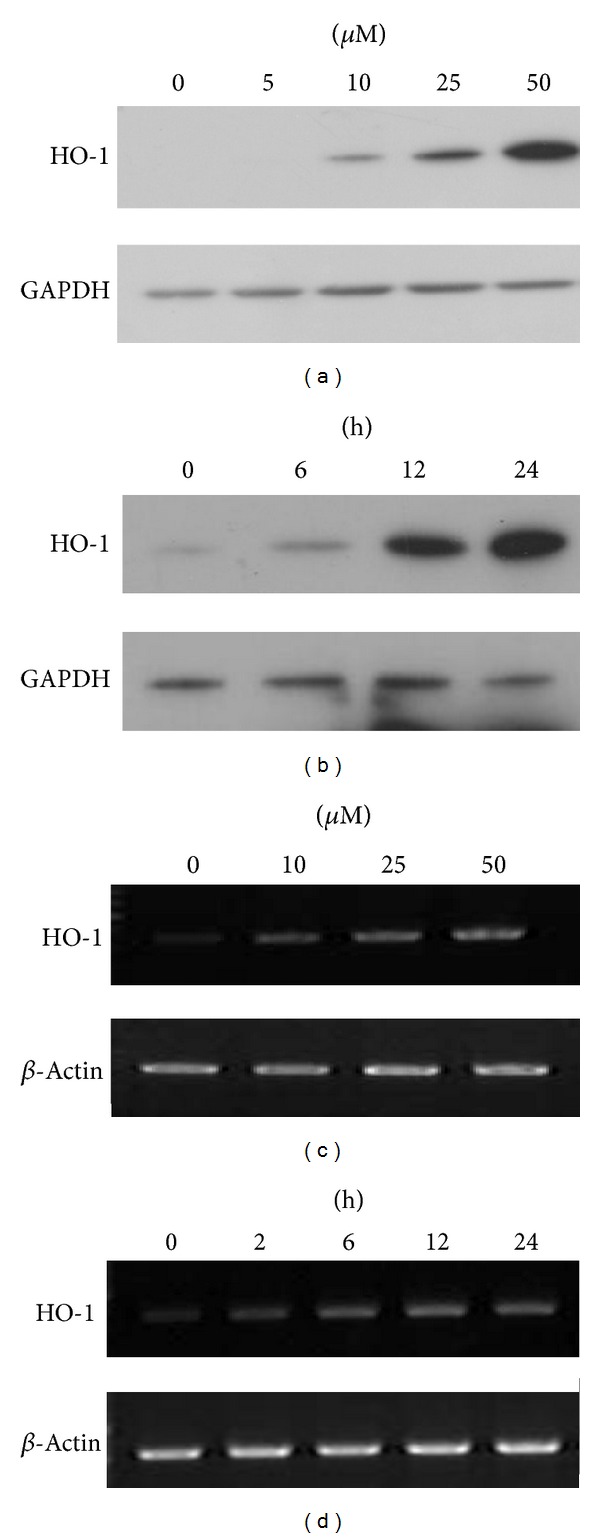
Induction of HO-1 by SPD in HUVECs. After treatment of HUVECs with various concentrations of SPD (a) at various time intervals (b), cell lysates were prepared, and 20 *μ*g samples of proteins were subjected to Western blotting using the anti-HO-1 antibody, and anti-glyceraldehyde 3-phosphate dehydrogenase (GAPDH) antibody as a loading control. Representative data from three independent experiments are shown. After treatment of HUVECs with various concentrations of SPD (c) at various time intervals (d), cell lysates were prepared. The total RNA was extracted and analyzed by RT-PCR. The amplified RT-PCR product was visualized on 1% agarose gel. Representative data from three independent experiments are shown.

Upregulation of HO-1 expression is thought to be a cellular stress response and to play an important role in protecting cells. Presently, we investigated the effect of various concentrations of SPD on HO-1 induction. Cells were incubated with the indicated concentrations of SPD for 24 h. SPD increased HO-1 protein expression in a concentration-dependent manner (Figure 1(a)). Treatment of cells with 50 *μ*M SPD also resulted in a time-dependent increase in HO-1 protein expression in HUVECs (Figure 1(b)). SPD increased the HO-1 mRNA level as well. After incubation with various concentrations of SPD for 8 h, the HO-1 mRNA level increased in a concentration-dependent manner (Figure 1(c)). SPD-treated HUVECs also showed increased HO-1 mRNA levels in a time-dependent manner (Figure 1(d)). 

### 3.2. Blockage of SPD-Induced HO-1 Protein Expression by Aminoguanidine, NAC, or PI3K/Akt Inhibitor

**Figure 2:**
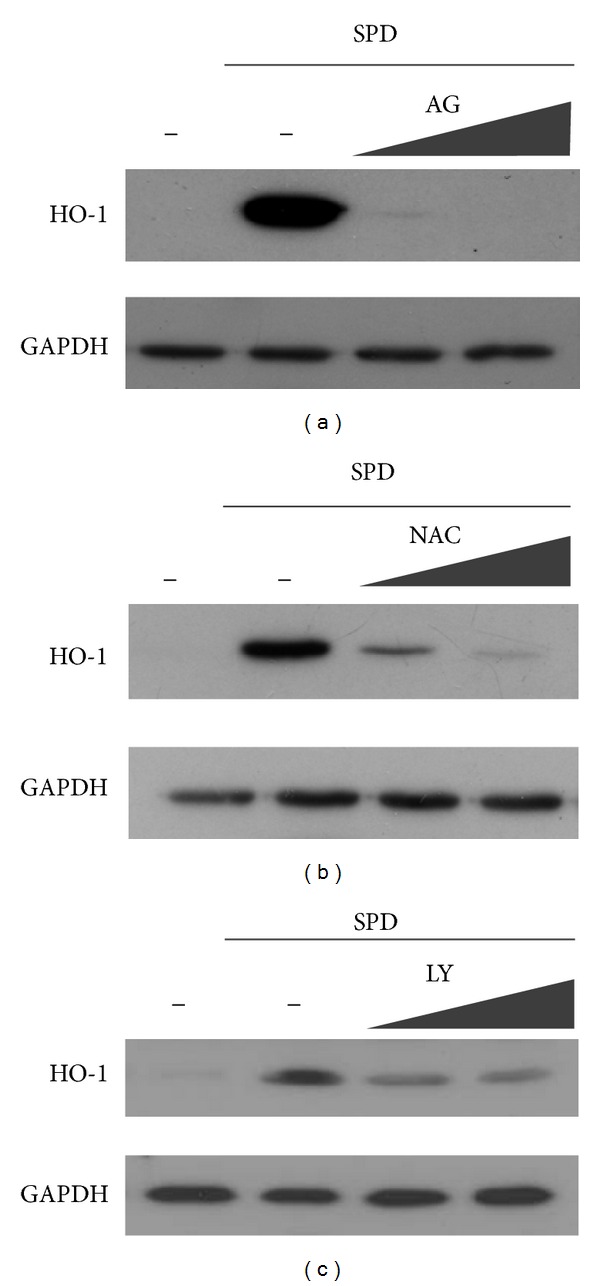
Blockage of SPD-induced HO-1 protein expression by aminoguanidine, NAC, or PI3K/Akt inhibitor. Cells were pre-treated with aminoguanidine (a), NAC (b), or LY 294002 (PI3K/Akt inhibitor) (c) 1 h prior to the treatment of SPD. After 24 h incubation, cell lysates were prepared, and 20 *μ*g samples of proteins were subjected to Western blotting, using anti-HO-1 antibody and anti-GAPDH.

Polyamines, including SPD, are deaminated by bovine serum amine oxidase under *in vitro* conditions [22], generating aldehydes, H_2_O_2_, and ammonia. The polyamine oxidation is inhibited by the serum amine oxidase inhibitor, aminoguanidine (AG) [23]. Therefore, we tested whether the deamination of SPD by oxidase in serum could affect HO-1 induction in human endothelial cells. Cells were pretreated with 0.1 or 1 mM AG and then treated with 50 *μ*M SPD. Induction of HO-1 by SPD was completely eliminated by the pretreatment of AG (Figure 2(a)). These results indicate the involvement of polyamine oxidation in the induction of HO-1 by SPD. 

The polyamine oxidation products are known to generate reactive oxygen species (ROS), the signaling molecules in mediating responses to various stimuli [24]. Since ROS have a role in the induction of HO-1 by several compounds [25, 26], we examined whether the HO-1 induction by SPD was dependent on ROS by using N-acetyl cysteine (NAC), a ROS scavenger. Pretreatment with NAC abolished HO-1 expression in SPD-stimulated HUVECs in a dose-dependent manner (Figure 2(b)). This implies the involvement of ROS in the induction of HO-1 by SPD in HUVECs.

To determine the upstream signaling pathway involved in SPD-mediated HO-1 induction, the effects of the specific inhibitors of the PI3K/Akt pathways on HO-1 expression were evaluated. Inhibitors of the PI3K/Akt pathways reduced SPD-induced HO-1 expression (Figure 2(c)). The observation supported a role for PI3K/Akt signaling in SPD-mediated HO-1 induction in HUVECs.

### 3.3. Nrf2 Nuclear Translocation and ARE-Luciferase Reporter Activity Induced by SPD

**Figure 3:**
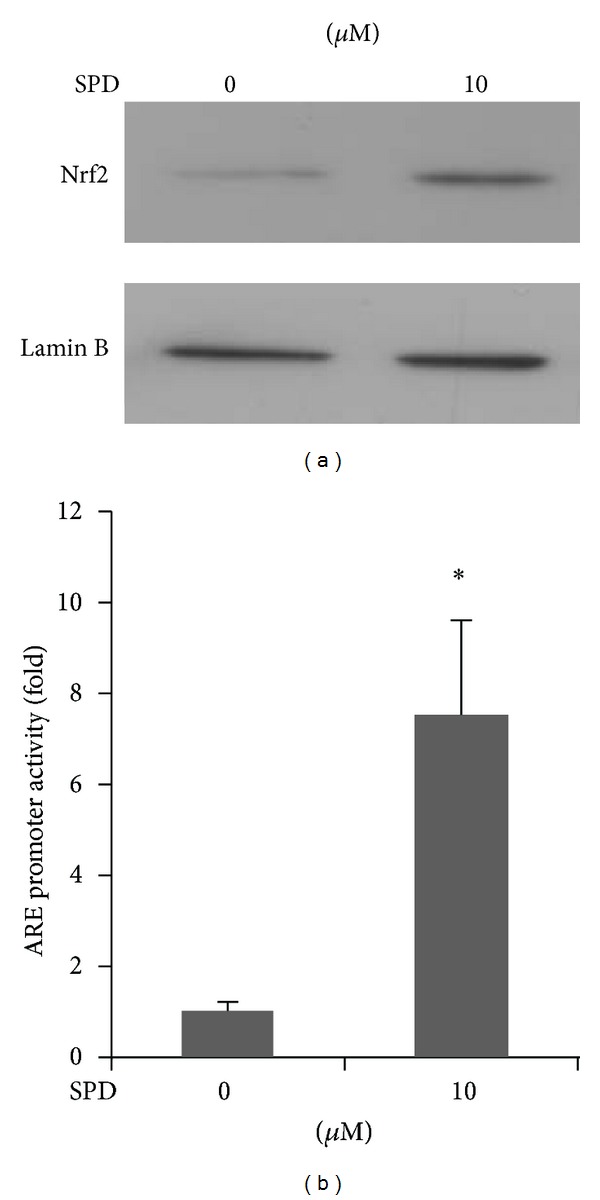
Nrf2 nuclear translocation and ARE-luciferase reporter activity induced by SPD. Cells were treated with SPD at the indicated concentration for 4 h. (a) Nuclear extracts were prepared, and 10 *μ*g samples of proteins were subjected to Western blotting, using an anti-Nrf2 antibody and an anti-Lamin B (a marker of nuclear protein) antibody. (b) Cells were transfected with an ARE-luciferase construct. After transfection, the cells were treated with 10 *μ*M SPD for 6 h, and the lysates were mixed with a luciferase substrate. A luminometer was used for measurement of luciferase activity. Data are the mean ± SD of quintuplicate experiments. *P < 0.05 versus control.

HO-1 belongs to a family of the genes encoding phase II detoxifying and antioxidant enzymes and is widely distributed in mammalian tissues. It is modulated by the Nrf2/Keap1 transcription factor system including Nrf2 binding to ARE in the nucleus [11]. Appropriately, we attempted to determine whether SPD could activate Nrf2 in association with HO-1 upregulation. For this, the cells were treated with 10 *μ*M of SPD for 4 h, and the nuclear fractions were extracted for the preparation of nuclear proteins. SPD treatment in HUVECs stimulated Nrf2 accumulation in the nucleus (Figure 3(a)). In addition to this, we measured ARE promoter activity using luciferase reporter system. Cells were transiently transfected with ARE luciferase reporter plasmids and treated with 10 *μ*M SPD for 6 h, and luciferase activity was determined. As expected, treatment with SPD at the smallest concentrations (10 *μ*M) increased ARE promoter activity by approximately 8 folds (Figure 3(b); *P < 0.05). These results indicate the association with Nrf2 activation in regulation of SPD-induced HO-1 expression in HUVECs.

### 3.4. Effect of SPD-Induced HO-1 Inhibition on Cell Death

**Figure 4:**
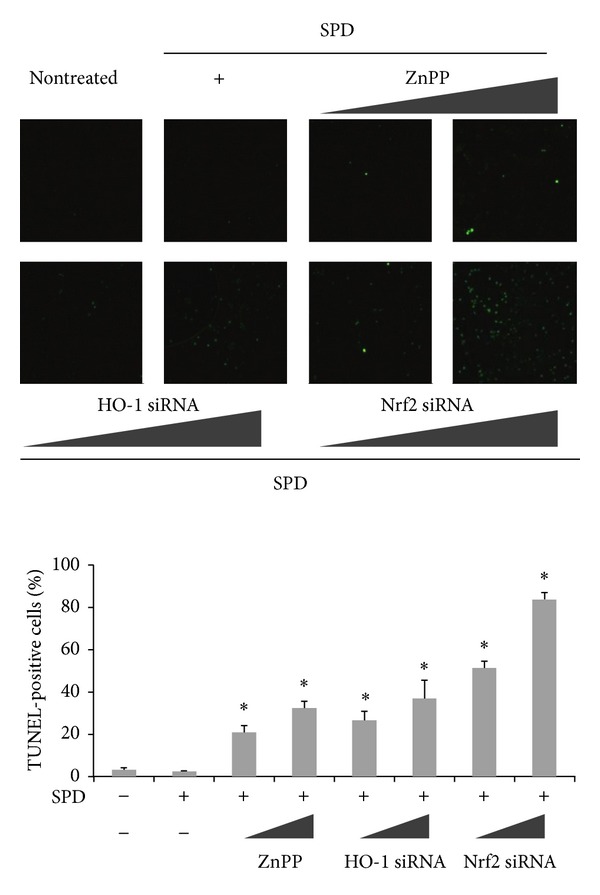
Effect of SPD-induced HO-1 inhibition on cell death. Cells were incubated in the absence or presence of ZnPP, siRNA of HO-1 or Nrf2 for 24 h before the indicated tests were performed. SPD-stimulated HUVECs were pretreated with or without ZnPP, HO-1 or Nrf2 siRNA. Protective effect of HO-1 induction on cell death was determined by *in situ* terminal nick-end labeling (TUNEL). Representative images illustrate fluorescent TUNEL (green) staining of cells cultured for 24 h before the indicated tests were performed. The percentage of TUNEL-positive cells per total cell count in each sample was calculated. At least 100 cells from three random fields were counted in each experiment. Data are the mean ± SD of triplicate experiments. *P < 0.05 versus control.

HO-1 is a well-known cytoprotective enzyme inhibiting cell death in different cell types [20, 27, 28]. To further determine whether the increased level of HO-1 enhanced by SPD confers cytoprotection, SPD-stimulated cells were pre-incubated with or without a specific HO-1, inhibitor, ZnPP, HO-1, or Nrf2 siRNA, and the presence of dead cells was assessed by *in situ* terminal nick-end labeling (TUNEL) staining, which is widely used in detecting DNA fragmentation *in situ*. SPD-treated cells showed little difference in nontreated cells, while pharmacological inhibition of HO-1, or the transcription factor Nrf2 resulted in a notable increase in the proportion of TUNEL-positive cells (Figure 4). These results suggest that HO-1 may exert a protective effect against SPD-stimulated cell death.

## 4. Discussion

The present study demonstrates that the polyamine SPD clearly induces HO-1 in human endothelial cells through activation of Nrf2. Presence of ROS scavenger or serum amine oxidase inhibitor abrogates the increase of HO-1. 

Although polyamines are essential for cell metabolism, the excessive accumulation within cells by exogenous addition of polyamine or high extracellular concentrations can result in toxic effects. Interconversion of polyamine is one of the ways to modulate polyamine pool homeostasis. Polyamines are synthesized from L-arginine via L-ornithine, or L-methionine by specific enzyme reactions. Putrescine is formed from L-ornithine by ornithine decarboxylase, and this combines with decarboxylated *S*-adenosylmethionine originating from L-methionine to produce spermidine in a reaction catalyzed by spermidine synthase [29]. Spermine is formed from spermidine by spermine synthase. Spermidine and spermine also undergo the retroconversion process by the action of spermidine/spermine *N*1-acetyltransferase and polyamine oxidase [30] and possibly spermine oxidase, in the direct conversion of spermine back to spermidine without acetylation [31]. During the catabolic process, toxic metabolites of polyamine are generated, including H_2_O_2_, ammonia, and aminoaldehyde(s). Moreover, after spermidine is oxidized by amine oxidase, an *α*, *β*-unsaturated aldehyde, acrolein, is formed by further spontaneous degradation [32]. Acrolein, which is highly reactive, can induce HO-1 expression as well as H_2_O_2_ [33] and ammonia [34]. Especially, the induction of HO-1 by toxic substances is thought to be an adaptive response [20].

HO-1 contributes to the cellular defense against oxidative stress generated by ROS. Increase of HO-1 activity protects cells from oxidative damage-induced cell death [21, 35]. In addition, since HO-1 is highly expressed in vascular tissues, it protects against vascular injury and confers cytoprotection in the circulation. The beneficial effect of HO-1 is mediated by the actions of its metabolic byproducts, carbon monoxide, and bilirubin. Carbon monoxide modulates blood fluidity and flow by regulating vasomotor tone, vascular smooth muscle cell proliferation, and platelet agglutination [36]. Bilirubin preserves endothelial cell integrity, prevents cell death, and increases vascular reactivity [37].

Transcriptional activation of the HO-1 gene is mediated by Nrf2 [38], which is initiated by translocation of Nrf2 into the nucles after electrophilic modification of Keap1 for dissociation of the Nrf2/Keap1 complex [11]. Nrf2 in nucleus binds to ARE in the promoter region of its target genes, the phase II detoxification and antioxidant genes, including NAD(P)H:quinone oxidoreductase 1, glutathione S-transferases, peroxiredoxin-1, and *γ*-glutamylcysteine ligase. Several oxidants induce the phase II detoxification and antioxidant gene expression via Nrf2 activation [39]. In particular, Nrf2 activation bestows cytoprotection to vascular tissues by induction of antioxidant gene expression and/or suppression of redox-sensitive inflammatory genes [40, 41]. The Nrf2/ARE pathway is involved in SPD-induced HO-1 expression, as some polyamines can indeed activate other Nrf2-related genes [42].

**Figure 5:**
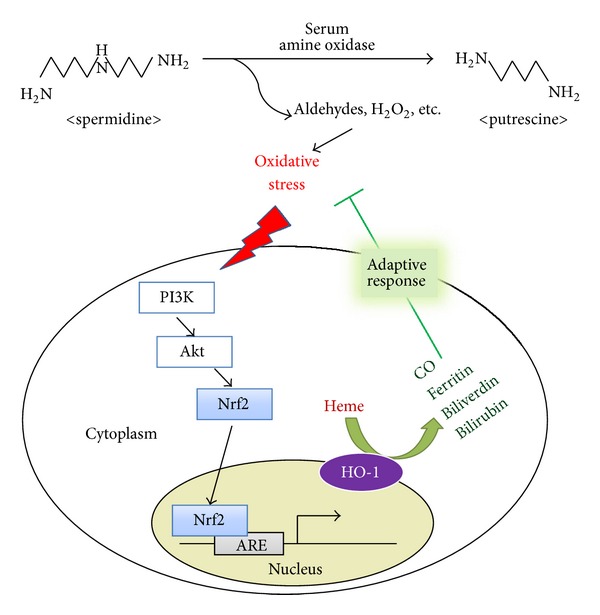
A scheme showing a proposed cytoprotective mechanism against SPD-induced oxidative stress by HO-1 induction. During the degradation of spermidine (SPD) to putrescine by serum amine oxidase, aldehydes, H_2_O_2_, and NH_3_ are produced, which induce oxidative stress. It triggers Nrf2 translocation and ARE binding through PI3K/Akt signaling. Nrf2 activation upregulates HO-1 expression. HO-1 catalyzed heme to biliverdin and bilirubin, with the concurrent release of bioactive molecules, such as ferritin and CO. Heme metabolites exert adaptive response and protect cells against SPD-induced oxidative stress.

In this study, we demonstrated that the polyamine SPD, possibly via its oxidized metabolites, induces HO-1 in human primary endothelial cells in association with Nrf2/ARE signaling system, and induction of HO-1 contributes to cell survival in oxidative stress-induced cell death (see Figure 5). This study provides evidence that the induction of HO-1 may have a role of cellular defense against the cytotoxic effects of SPD oxidation in vascular endothelial cells.
